# Calibrating Transcriptional Activity Using Constitutive Synthetic Promoters in Mutants for Global Regulators in *Escherichia coli*

**DOI:** 10.1155/2018/9235605

**Published:** 2018-03-21

**Authors:** Ananda Sanches-Medeiros, Lummy Maria Oliveira Monteiro, Rafael Silva-Rocha

**Affiliations:** Systems and Synthetic Biology Lab, FMRP - University of São Paulo, Ribeirão Preto, SP, Brazil

## Abstract

The engineering of synthetic circuits in cells relies on the use of well-characterized biological parts that would perform predicted functions under the situation considered, and many efforts have been taken to set biological standards that could define the basic features of these parts. However, since most synthetic biology projects usually require a particular cellular chassis and set of growth conditions, defining standards in the field is not a simple task as gene expression measurements could be affected severely by genetic background and culture conditions. In this study, we addressed promoter parameterization in bacteria in different genetic backgrounds and growth conditions. We found that a small set of constitutive promoters of different strengths controlling a short-lived GFP reporter placed in a low-copy number plasmid produces remarkably reproducible results that allow for the calibration of promoter activity over different genetic backgrounds and physiological conditions, thus providing a simple way to set standards of promoter activity in bacteria. Based on these results, we proposed the utilization of synthetic constitutive promoters as tools for calibration for the standardization of biological parts, in a way similar to the use of DNA and protein ladders in molecular biology as references for comparison with samples of interest.

## 1. Introduction

Understanding the logic underlying the genetics of a microorganism based on the dynamics of its promoters and transcription factors is essential for manipulation of other living systems. A way to study this logic is introducing synthetic circuits provided with a reporter gene into living cells and analyzing the results of the expression [1, 2]. However, the success of the implementation of complex circuits in living cells relies strongly on the correct production of the molecular components of the cells and is not limited to the influences of the promoters and transcription factors on gene expression. Several factors are responsible for controlling gene expression, including the rates of mRNA and protein production and their rates of degradation. However, synthesis of mRNA depends strongly on promoter strength, which determines how frequently the RNA polymerase (RNAP) is recruited to the promoter to initiate transcription [3]. On the other hand, the rate of protein production depends strongly on the strength of the ribosome binding site (RBS) in recruiting ribosomes for the translation of the target protein [4]. Additionally, the dilution or degradation of mRNA and proteins depends on the physiological state of the cell just as how their synthesis also relies on cell physiology with respect to the availability of nucleotides, amino acids, RNAP, and ribosomes [5]. In this way, changes in cell physiology and growth conditions can cause variability in gene expression in a manner that is independent of promoter regulation [5].

On account of these possible variations between the cells, several attempts have been made to establish biological standards for promoter activity, and the use of internal promoters as references has been proposed some years ago [6, 7]. More recently, the use of calibrated internal promoters has been proposed as an alternative for defining relative promoter activities during experimental measurements of transcription levels. In this method, an endogenous (or reference) promoter is placed in the same plasmid as the target promoter, each of them controlling the expression of a different fluorescent protein, and the intrinsic promoter activity is calculated as a ratio of the two outputs [8]. However, the expression of additional genes in the host bacterium can increase genetic load and influence gene expression as well. In this way, inserting a calibrated internal promoter would disturb cell functions [9]. Additionally, most methods have focused on the analysis of maximal promoter activity at fixed conditions or on linear expression range of promoter activity, limiting the utilization of standards on condition where cells are subjected to changing physiological regimens [8]. These requirements make the use of calibration methods for the analysis of regulated promoters extremely difficult.

In this study, we seek to analyze intrinsic promoter activity using a single reporter gene in different strains *of Escherichia coli* by using a simple and straightforward protocol. For the determination of intrinsic promoter activity, we used a low-copy number plasmid based on the p15a origin of replication (ori) and a short-lived GFP with LVA tag [10]. We analyzed four constitutive promoters available in the Registry of Standard Biological Parts and a wild-type *Plac* promoter as regulated system. As hosts, we used two strains of *E. coli* with mutant global regulatory proteins, *ihf* and *fis*, which are responsible for regulating the expression of hundreds of genes in this bacterium [11], obtained from the widely used Keio collection of *E. coli* mutants [12]. Additionally, glucose was used as the external source of variation, since all strains exhibited improved growth rates in its presence. Under the conditions of the analysis, we observed that the system we had used exhibited invariant promoter activities that were independent of the strains and growth conditions used, indicating it was able to demonstrate the intrinsic properties of the promoters analyzed. In addition, to prove that our calibrator works, we tested the natural promoter of *Pseudomonas putida Pm* promoter with different concentrations of 3-methylbenzoate (3MBz) [13] and calibrate it with our four constituent promoters in liquid and solid medium. In this way, it was possible to verify that the calibrator works and presents a potential application in synthetic biology. In this regard, we propose a simple, plasmid-based and single reporter method for promoter calibration that is compatible for use with regulated promoters and changes in growth conditions, which could be fundamental to the characterization of biological parts in synthetic biology.

## 2. Material and Methods

### 2.1. Bacterial Strains, Plasmids, and Growth Conditions

The bacterial strains, plasmids, and primers used in this study are listed in Table 1. *E. coli* DH5*α* was used for cloning the pMR1-*Pjx* (where *x* stands for 100, 106, 114, and 113) and pMR1-*Plac* vectors [14] by transformation using the heat-shock method, and *E. coli* DH10B was used for cloning pGLR2-*Pjx* vectors by electroporation and for GFP/Lux expression analysis [15]. For the calibration of promoter activity, *E. coli* BW25113 was used as wild-type strain, and *ihf* (*Δihf*) or *fis* (Δ*fis*) mutants (from the Keio collection) were used as mutant hosts with reduced growth rate. Plasmids pMR1 and pGLR2 were used as reporter systems for promoter analysis. Plasmid pMR1 has a low-copy p15a ori, a chloramphenicol-resistance marker, and two genes encoding fluorescent proteins oriented in opposite directions (*mCherry* and *gfplva*). Plasmid pGLR2 has a low-copy RK2 origin of replication, a kanamycin resistance marker, and two reporter genes oriented in the same direction, namely, the GFP gene followed by *luxCDABE*. Although the vector has GFP, in this work when pGLR2 was used, only the Lux was measured. The *E. coli* strains were grown at 37°C with aeration at an agitation rate of 180 rpm in LB medium (for overnight growth) or M9 minimal medium (containing 6.4 g/L Na_2_HPO_4_•7H_2_O, 1.5 g/L KH_2_PO_4_, 0.25 g/L NaCl, and 0.5 g/L NH_4_Cl) supplemented with 2 mM MgSO_4_, 0.1 mM CaCl_2_, 0.1 mM casamino acids, and 1% glycerol as the sole carbon source (for growth during the analysis). When required, chloramphenicol (34 *μ*g/mL), kanamycin (50 *μ*g/mL), or glucose (0.4%) was added to the medium. In the minimal medium, the antibiotics were added at half of the previously mentioned concentrations.

### 2.2. Plasmid and Strain Construction

For the experiments, oligonucleotides were synthesized (*Exxtend*, Campinas, Brazil) based on the synthetic constitutive promoters from the iGEM BBa_J23104 set of promoters (http://parts.igem.org/Part:BBa_J23104), with an annealing site on pMR1 and restriction sites for *EcoRI* and *BamHI*. The promoters J23100, J23106, J23114, and J23113 (referred here as *Pj100*, *Pj106*, *Pj114*, and *Pj113*, resp.) were used (see Table 1). Once these fragments were amplified by PCR, they were digested by *EcoRI* and *BamHI* and inserted into the multiple cloning sites (MCS) of pMR1 and pGLR2, and thus, generating pMR1-*Pjx* and pGR2-*Pjx*. These plasmids were inserted into DH5*α* and DH10B strains, respectively, cloned, and sequenced. The plasmids, pMR1-*Pjx* were inserted into *E. coli* BW25113 and into *ihf* and *fis* mutants obtained from the Keio collection.

The *xylS*, *PxylS*, and *Pm* promoters were PCR amplified with Phusion High-Fidelity DNA polymerase (Thermo Fisher Scientific) using the primer pairs 5_xylS_EcoRI (5′-GAA TTC TCA AGC CAC TTC CTT TTT GCA TTG-3′) and 3_Pm_BamHI (5′-GGA TCC ATT ATT GTT TCT GTT GCA TAA AGC C-3′) and pSEVA438 vector (pBBR1 replication origin, Sm/Sp; Silva-Rocha and de Lorenzo [1]) as template. These primers introduced EcoRI and BamHI restriction sites (underlined) at the 5′ and 3′ ends, respectively. The PCR products were gel purified, digested with EcoRI/BamHI, and ligated to the pMR1 vector previously cut with the same restriction enzymes. The resulting plasmids were sequenced to check integrity and inserted to *E. coli* strains (*E. coli* BW25113and into *ihf* and *fis* mutants). The resulting plasmid was named pMR1-xylS-*Pm.*

### 2.3. GFP Fluorescence and Bioluminescence Assays and Data Processing

To analyze promoter activity, single colonies of recombinant strains containing pMR1-*Pjx* and *E. coli* DH10B containing pGLR2-*Pjx* were grown overnight in LB medium that was supplemented with chloramphenicol (34 *μ*g/mL) or kanamycin (50 *μ*g/mL) for plasmid selection at 37°C with aeration and agitated at 180 rpm. The strains grown overnight were washed with MgSO_4_ (10 mM) buffer, resuspended in the same buffer, and diluted to a ratio of 1 : 20 with M9 minimal medium (containing 6.4 g/L Na_2_HPO_4_•7H_2_O, 1.5 g/L KH_2_PO_4_, 0.25 g/L NaCl, and 0.5 g/L NH_4_Cl) supplemented with 2 mM MgSO_4_, 0.1 mM CaCl_2_, 0.1 mM casamino acids, chloramphenicol (17 *μ*g/mL), and 1% glycerol as the sole carbon source. When required, glucose (0.4%) was supplemented to the medium. In total, 200 *μ*L of the culture was placed in a 96-well plate and analyzed using a *Victor X3* plate reader (*PerkinElmer*) over several hours at 37°C. At 30-minute time intervals, the optical density at 600 nm (OD_600nm_) and the fluorescence (excitation 485 nm and emission 535 nm) were measured for the strains containing pMR1-*Pjx*; the optical density at 600 nm (OD_600nm_), the fluorescence (excitation 485 nm and emission 535 nm), and the bioluminescence were measured for DH10B containing pGLR2-*Pjx*. Promoter activities were expressed as fluorescence or bioluminescence normalized by the OD_600nm_ upon background normalization (fluorescence/OD_600nm_). As a positive control for pMR1-*Pjx* analysis, wild type *lac* promoter (*Plac*), which is regulated by CRP, was used. Data analysis and representation was performed using *Microsoft Excel (2016)* and ad hoc *R* script. To prove that the pMR1-*Pjx* system works as a gene expression standard, the natural promoter of *P. putida Pm*, which is regulated by *xylS* when this regulator is induced by 3MBz, was used [13]. The pMR1-xylS-*Pm* construct contains the *xylS* promoter (*PxylS*), which in the presence of 3MBz leads to the expression of the XylS regulatory protein. The XylS regulator binds to 3MBz and activates the *Pm* promoter by inducing the expression of GFP. Data analysis and representation were performed using ad hoc *R* script.

## 3. Results and Discussion

### 3.1. Quantification of Constitutive Promoter Activity Using GFP and *luxCDABE* Reporters

**Figure 1:**
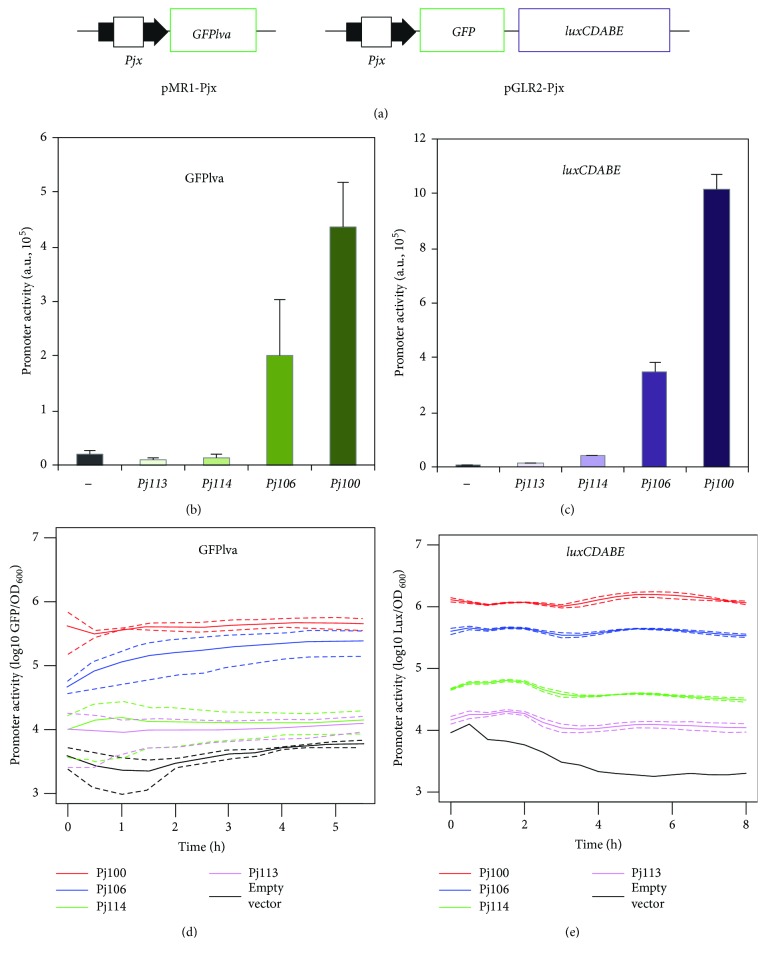
Construction and validation of the reporter systems. (a) Synthetic promoters were cloned into the plasmid pMR1, which contains a short-lived GFPlva variant, and pGLR2, a broad host range vector containing a GFP-*luxCDABE* reporter system. (b) Maximal promoter activity of the four promoters in pMR1 vector. (c) Maximal promoter activity analyzed by monitoring lux expression using pGLR2 constructions. (d) GFP expression profile along the growth curve from reporters cloned in pMR1 vector. (e) *lux* expression profile along the growth curve from reporters cloned in pGLR2 vector. The solid lines represent the average values calculated using data from three independent experiments while dashed lines represent standard deviation from the samples.

For the analysis of promoter activities, we used two reporter plasmids based on a short-lived GFP variant placed into a narrow-host-range vector (pMR1 [14]) and a synthetic GFP-*luxCDABE* reporter system placed into a broad-host-range vector [15, 16] to measured Lux, as represented in Figure 1(a). In order to observe the effects of these differences (regarding use of different reporter systems) on the measurement of the activities of the promoters of interest, we analyzed the promoter activities of four BioBrick parts, namely Pj100, Pj106, Pj114, and Pj113, that contain mutations in the sequences at −35 or −10 and exhibit about 100%, 50%, 10%, and 1% activities, respectively (relative to *Pj100* activity). As shown in Figures 1(b) and 1(c), maximal promoter activity of the four synthetic promoters analyzed were virtually identical for both the short-lived GFPlva and the *luxCDABE* reporters, resulting in relative activity values that are closer to the expected value. When we analyzed the promoter activities during the growth period of *E. coli* using the GFPlva and Lux reporters, we observed that the differences were present throughout the growth period of the bacteria, with better differentiation of the intrinsic promoter activities achieved using the luminescent reporter system (Figures 1(d) and 1(e)). Although the luciferase reporter provided better differentiation, GFP reporter allows uses to perform single-cell experiments that cannot be made using light-emitting reporters. Since most synthetic biology works use the GFP reporter, and moreover, GFP provided sufficient resolution to analyze the promoters and also allowed for single-cell analysis (a possible calibrator approach), we focused on the section on the pMR1 reporter system containing the short-lived variant of the reporter protein.

### 3.2. Robust Calibration of Promoter Activities under Different Pleiotropic and Growth Conditions

**Figure 2:**
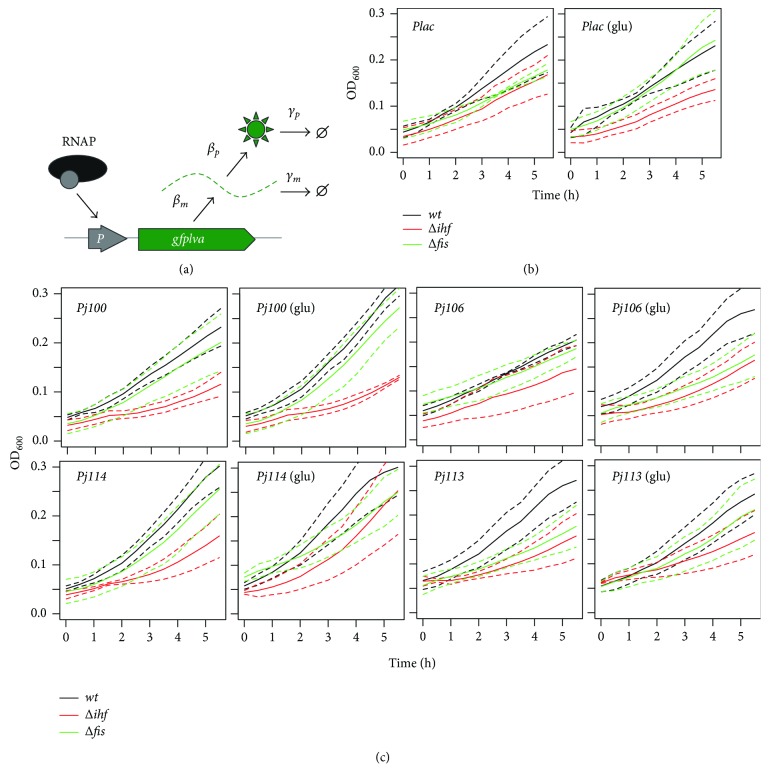
Quantification of growth variation in different *E. coli* strains under two physiological regimens. (a) Schematic representation of the main steps for gene expression in bacteria. The strength of the interaction between RNA polymerase (RNAP) and target promoter determines the rate of mRNA synthesis (*β_m_*), while the RBS sequence determines the rate of protein translation (*β_p_*). The rates of mRNA and protein dilution or degradation (*γ_m_* and *γ_p_*, *resp.*) depends on cell growth and physiological regimens of the cells. (b) Growth curve of *E. coli* strains harboring a *Plac::GFPlva* fusion in minimal medium with 1% glycerol (left) or 1%glycerol plus 0.4% glucose (right) as carbon source. (c) Growth curve of *E. coli* strains harboring different promoter fusions (*Pj100*, *Pj106*, *Pj114*, and *Pj113*) in minimal medium with 1% glycerol or 1%glycerol plus 0.4% glucose (labeled as glu) as carbon source. Solid lines represent average values calculated using data from three independent experiments for wild type (black), *Δihf* (red), and *Δfis* (green) strains, while dashed lines represent the upper and lower limits of standard deviations.

During the experiments, wild type and mutant strains of *E. coli* were grown in minimal M9 medium with 1% glycerol and 1% glycerol plus 0.4% glucose in order that the cells were adapted to a richer physiological regimen.  Figure 2(a) represents the critical steps in gene expression that were influenced by the bacterial hosts and growth conditions used. In this regard, the rate of mRNA synthesis (*βm*) was the main parameter controlled by a specific synthetic promoter, while the rate of protein synthesis (*βp*) was dependent on the strength of the RBS involved (which was the same in all constructs analyzed). Additionally, the rates of mRNA and protein degradation were dependent on the nature of the reporter sequence (i.e., due to differences in the sequence of reporter genes) and the growth rate of the bacteria (since fast-growing bacteria have higher dilution rates of mRNA and protein than slow-growing bacteria). In this method, the use of constitutive promoters with different strengths allowed for variations in *βm* and facilitated the analysis of its sensitivity to changes in the dilution or degradation rates of mRNA and proteins. As shown in Figures 2(b) and 2(c), both *ihf* and *fis* mutants exhibited reduced growth compared to the wild type for all constructs analyzed. However, in all cases, the addition of 0.4% glucose to the growth medium resulted in a stepwise improvement in the growth of the strains. In other words, in the presence of glucose, bacterial growth is faster, although there is no glucose effect on the final promoter activity. Our calibrator approach is an interesting way to avoid the genetic background differences, indicating that the calibrator can be used in several conditions to standardize promoter studies using different strains under a growth condition variety (glucose or 3MBz—performed below).

**Figure 3:**
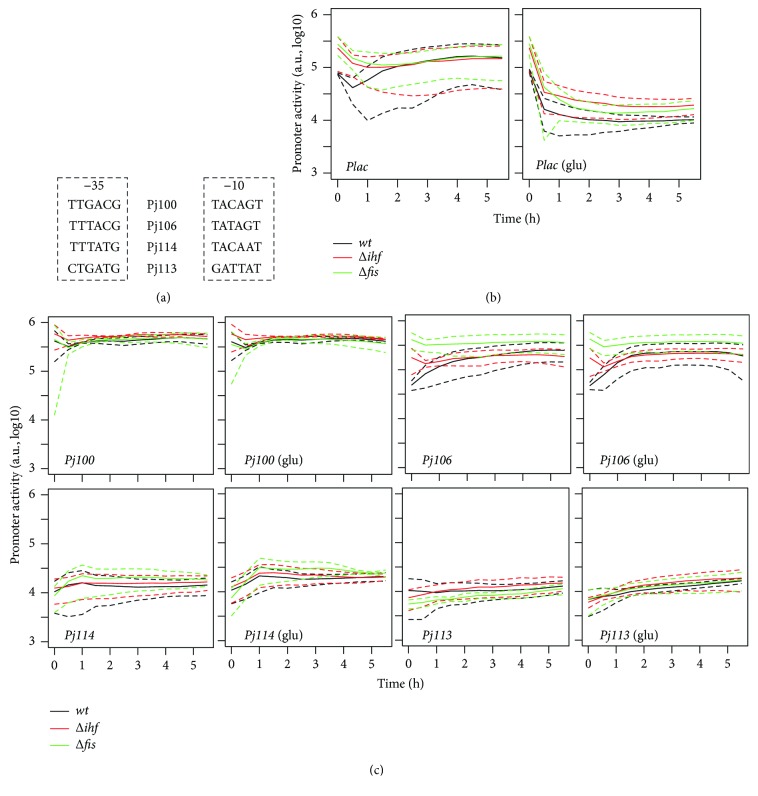
Quantification of promoter activities in different *E. coli* strains. (a) Representation of the sequences at −10 or −35 for the four constitutive promoters analyzed, using bold letters for bases conserved related to *Pj100* reference. (b) Promoter activity of *E. coli* strains harboring a *Plac::GFPlva* fusion in minimal medium with 1% glycerol (left) or 1%glycerol plus 0.4% glucose (right) as carbon source. (c) Promoter activity of *E. coli* strains harboring different promoter fusions (*Pj100*, *Pj106*, *Pj114*, and *Pj113*) in minimal media with 1% glycerol or 1%glycerol plus 0.4% glucose (labeled as glu) as carbon source. Solid lines represent the average values calculated using data from three independent experiments for wild type (black), *Δihf* (red), and *Δfis* (green) strains, while dashed lines represent the upper and lower limits of standard deviations.

In order to observe the effects of the differences in strains and growth medium on the measurements of the activities of the promoters of interest, we analyzed four synthetic promoters that contain sequence differences at −35 or −10 (Figure 3(a)). As shown in Figure 3(b), the regulated *Plac* promoter (a natural promoter used as reference) exhibited strong activity in the three strains analyzed (wild type, *Δihf*, and *Δfis*), and this activity was fully suppressed in the presence of glucose (due to the inactivation of CRP [17]). When we analyzed promoter activity of the four synthetic promoters, we observed that the promoter dynamics and steady state promoter activity were almost invariant in the different mutant strains and under the two growth conditions (Figure 3(c)), indicating that these promoters were not influenced by the drastic physiological variations regarding the different strains of *E. coli* (*E. coli* BW25113, and into *ihf* and *fis* mutants). It is noteworthy that the same was observed for the addition of glucose to the medium that did not compromise the promoter activity. Again showing that the internal calibrator can be used in different situations. When we performed a comparison of the observed promoter activities, *Pj106* exhibited an activity level very close to the expected value (45.7% observed versus 47% expected), whereas *Pj114* and *P113* exhibited promoter activities varying by~ 3% and 2.3%, respectively, from the value of the activity exhibited by *Pj100* (compared to the expected values, 10% and 1%, resp.). These expected values come from the previous analysis made by iGEM BBa_J23104 set of promoters (http://parts.igem.org/Part:BBa_J23104). These differences are due to the differences in the reporter, plasmids, and strains used for promoter characterization. Additionally, the *Plac* promoter exhibited activity of value about 30% under nonrepressive conditions when compared to *Pj100* reference promoter.

These results show that the use of the short-lived GFP reporter in combination with constitutive promoters is a simple way to calibrate promoter activity under user-specific experimental conditions, similar to the way that DNA and protein ladders are used in molecular biology techniques as references for the comparison of specific targets. In conclusion, our data shows how intrinsic promoter activity can be calibrated using single reporter genes and simple data processing without the need for using internal promoter references.

### 3.3. The Calibrator Can Be Applied to the Induction System xylS-*Pm*

**Figure 4:**
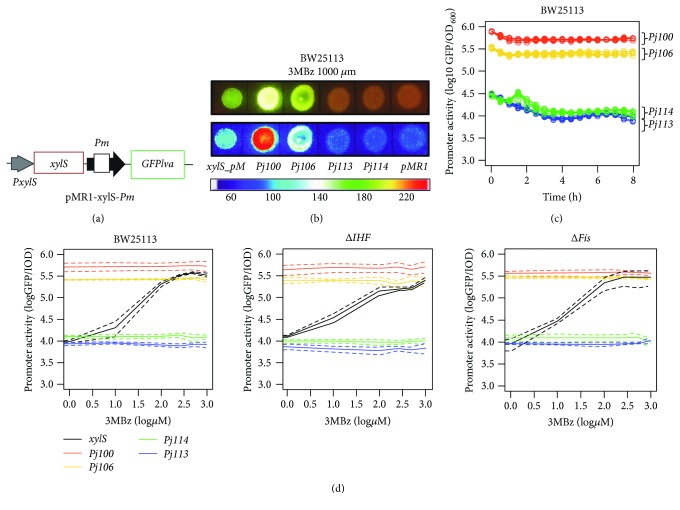
The calibrator can be applied to the induction system xylS-*Pm*. (a) xylS promoters (*PxylS*), xylS protein, and *Pm* promoter were cloned into the plasmid pMR1, which contains a short-lived GFP variant. (b) xylS-*Pm* calibration in LB solid medium with 1000 *μ*M of 3MBz added. This calibration was performed in BW25113 *wt* strains. (c) *Pjx* promoter activity analyzed by 8 hours of experiment by monitoring GFPlva expression using pMR1 constructions. (d) GFP expression profile for 7 different 3MBz concentrations for *Pjx* and xylS-*Pm* in pMR1 vector, 4.5 hours after the induction. Solid lines represent the average values calculated using data from three independent experiments for wild type, *Δihf*, and *Δfis* strains, while dashed lines represent the upper and lower limits of standard deviations.

In order to prove that the set of four promoters proposed in this work acts as an internal calibrator even when applied to an induced expression system, we analyzed the four synthetic promoters and pMR1-xylS-*Pm* system (Figure 4(a)) with increasing concentrations of 3MBz. In order to demonstrate that our calibrator is robust and even works in a blue light transilluminator, we analyzed colonies grown on petri dish contend medium LB plus 3MBz 1000 *μ*M. As shown in Figure 4(b), pMR1-xylS-*Pm* displayed the same promoter activity as pMR1-P106. Next, we tested the system at increasing 3MBz inductor concentrations; they were carried out on the three *E. coli* strains previously used in this work. The data shown in Figure 4(d) are relative to 4.5 hours after the start of the induction. From Figure 4, it is possible to note that the increasing concentration of 3MBz did not promote differences in *Pjx* promoter activity (Figure 4(d)), neither during the 8 hours of the experiment (Figure 4(c)). On Figure 4(c), it is important to note that each color on the graph represents a different *Pjx* synthetic promoter and the set of lines belonging to each color group refers to different 3MBz concentrations. In this sense, it is possible to note that there are no differences between the set color lines. This result suggests that the 3MBz addition do no promote differences on promoter activity. On the other hand, for a 3MBz-induced system, the aromatic compound produced a change in promoter activity for a sigmoidal curve, proportional to the 3MBz increase concentration (Figure 4(d)).

A brief and simple conclusion can be made from Figure 4(d), regardless of the host strain used, in the range of 1 to 10 *μ*M (0 to 1 on the x axis) concentration, the *Pm* promoter presents similar promoter activity to *Pj114* promoter. On the other hand, in the range of 10 to 100 *μ*M (1 to 2 on the x-axis) concentration, *Pm* presents intermediate promoter activity to *Pj114* and *Pj106* promoters. Finally, in the range of 100 to 1000 *μ*M (2 to 3.0 on the *x*-axis) concentrations, *Pm* presents promoter activity close to *Pj106* promoter activity. Additionally, we can safely confirm that the sigmoidal form for the xylS-*Pm* system is due to the 3MBz addition and not by environmental or host changes, since the calibration system does not change under these conditions.

## 4. Conclusions

The standardization of biological parts for the construction of complex circuits forms the basis of synthetic biology [18–21]. In this regard, failure in the implementation of constructed synthetic biological circuits may occur when poorly characterized parts are used, and several strategies have been proposed to mitigate this problem [6, 22–24]. In this report, we have highlighted that simple experimental techniques involving the use of a single fluorescent reporter and plasmids are sufficient to provide robust characterization of transcriptional elements without the necessity of using of dual markers and complicated mathematical treatments [8]. Additionally, plasmids provide an easy way of implementing synthetic circuits that accelerates design-build-test cycles in synthetic biology. Once a circuit has been effectively implemented and tested, the introduction of a single copy of the construct by using a chromosome insertion on a same region for all promoters is recommended in order to enhance the performance of the system as well as provide stable strains for final use because an insertion on different regions could modify the GFP expression [25–27]. In this sense, the use of this promoter on different chromosome regions could provide a way to standardize the variation of gene expression caused by variations on chromosome position and structure. At the same time, the use of low-copy number plasmids can provide similar results as can the use of single-copy set-ups under certain circumstances [1, 15]. In general, since each synthetic biology project has its own design and uses specific hosts and experimental conditions, the use of calibrators such as those described in this paper could provide a simple way to standardize the experimental conditions used. This would be similar to the use of molecular-weight size markers in molecular biology techniques as references for the comparison of samples of interest under varying experimental conditions. Although the calibration methods used in this study were implemented in strains of the Keio collection of *E. coli* mutants [12], we expect that the validations presented here will be adopted by other research groups studying synthetic biology as well as molecular microbiology. Additionally, the approaches used in this research study can be easily adopted using alternative plasmid standards for gram negative bacteria other than *E. coli* such as the vectors available at the SEVA database [16], thus creating a significant impact on research in microbiology.

## Figures and Tables

**Table 1:** Strains, plasmids and primers used in this study.

Strains, plasmids, and primers	Description	Reference
Strains		
*E. coli DH5α*	F^−^* endA1 glnV44 thi-1 recA1 relA1 gyrA96 deoR nupG purB20 φ*80d*lacZ*ΔM15 Δ(*lacZYA-argF*)U169, hsdR17(*r_K_*^−^*m_K_*^+^), *λ*^−^.	[12]
*E. coli DH10B*	*mcrA Δmrr-hsdRMS-mcrBC) φ 80lacZΔM15 ΔlacX74 recA1 araD139 Δ (ara-leu)7697 galU galK rpsL endA1 nupG Δdcm.*	[12]
*E. coli BW25113*	*lacI* ^+^ *rrnB*_T14_ Δ*lacZ*_WJ16_* hsdR*514 Δ*araBAD*_AH33_ Δ*rhaBAD*_LD78_* rph-1*	[12]
	Δ*(araB–D)567* Δ*(rhaD–B)568* Δ*lacZ4787*(::*rrnB-3*) *hsdR514 rph-1.*	
*E. coli JW1702*	*E. coli BW25113 Δihf* mutant	[12]
*E. coli JW3229*	*E. coli BW25113 Δfis* mutant	[12]
Plasmids		
pMR1	Cm^R^, *ori p15a*. GFPlva promoter probe vector	[14]
pMR1-*Pj113*	pMR1 with *Pj113* cloned as EcoRI/BamHI fragment	This work
pMR1-*Pj114*	pMR1 with *Pj114* cloned as EcoRI/BamHI fragment	This work
pMR1-*Pj106*	pMR1 with *Pj106* cloned as EcoRI/BamHI fragment	This work
pMR1-*Pj100*	pMR1 with *Pj100* cloned as EcoRI/BamHI fragment	This work
pMR1-*Plac*	pMR1 with *Plac* promoter cloned as EcoRI/BamHI fragment	[28]
pGLR2	Km^R^, *oriT*, *ori* RK2. SEVA-based vector with dual GFP-*lux* reporter	[15]
pGLR2-*Pj113*	pGLR2 with *Pj113* cloned as EcoRI/BamHI fragment	This work
pGLR2-*Pj114*	pGLR2 with *Pj114* cloned as EcoRI/BamHI fragment	This work
pGLR2-*Pj106*	pGLR2 with *Pj106* cloned as EcoRI/BamHI fragment	This work
pGLR2-*Pj100*	pGLR2 with *Pj100* cloned as EcoRI/BamHI fragment	This work
pMR1-*xylS-Pm*	pMR1 with *PxylS, xylS* and *Pm* cloned as EcoRI/BamHI fragment	This work
Primers		
Pj100-FW	GAATTCTTGACGGCTAGCTCAGTCCTAGG	This work
Pj100-RV	TACAGTGCTAGCAAGTGGATCCTTGCGATC	This work
Pj106-FW	GAATTCTTTACGGCTAGCTCAGTCCTAGGTA	This work
Pj106-RV	TAGTGCTAGCAAGTGGATCCTTGCGATC	This work
Pj114-FW	GAATTCTTTATGGCTAGCTCAGTCCTAGGT	This work
Pj114-RV	ACAATGCTAGCAAGTGGATCCTTGCGATC	This work
Pj113-FW	GAATTCCTGATGGCTAGCTCAGTCCTAGGG	This work
Pj113-RV	ATTATGCTAGCAAGTGGATCCTTGCGATC	This work
5_xylS_EcoRI	GAATTCTCAAGCCACTTCCTTTTTGCATTG	This work
3_Pm_BamHI	GGATCCATTATTGTTTCTGTTGCATAAAGCC	This work
